# Concept for the Evaluation of Carcinogenic Substances in Population-Based Human Biomonitoring

**DOI:** 10.3390/ijerph19127235

**Published:** 2022-06-13

**Authors:** Klaus-Michael Wollin, Petra Apel, Yvonni Chovolou, Ulrike Pabel, Thomas Schettgen, Marike Kolossa-Gehring, Claudia Röhl, on behalf of the Human Biomonitoring Commission of the German Environment Agency

**Affiliations:** 1 Formerly Public Health Agency of Lower Saxony, 30449 Hannover, Germany; klaus-michael.wollin@t-online.de; 2 German Environment Agency (UBA), 14195 Berlin, Germany; petra.apel@uba.de (P.A.); marike.kolossa@uba.de (M.K.-G.); 3 North Rhine-Westphalia Office of Nature, Environment and Consumer Protection, 45659 Recklinghausen, Germany; yvonni.chovolou@lanuv.nrw.de; 4 German Federal Institute for Risk Assessment (BfR), 10589 Berlin, Germany; ulrike.pabel@bfr.bund.de; 5 Institute for Occupational, Social and Environmental Medicine, Medical Faculty, RWTH Aachen University, 52074 Aachen, Germany; tschettgen@ukaachen.de; 6 Department of Environmental Health Protection, State Agency for social Services (LAsD) Schleswig-Holstein, 24534 Neumünster, Germany; 7 Institute of Toxicology and Pharmacology for Natural Scientists, Christiana Albertina University of Kiel, 24105 Kiel, Germany

**Keywords:** human biomonitoring, carcinogens, risk assessment, internal exposure

## Abstract

The Human Biomonitoring (HBM) Commission at the German Environment Agency holds the opinion that for environmental carcinogens for which no exposure levels can be assumed and are harmless to health, health-based guidance values corresponding to the classical definition of the HBM-I or HBM-II value cannot be established. Therefore, only reference values have been derived so far for genotoxic carcinogens from exposure data of the general population or subpopulations. The concept presented here opens up the possibility of performing health risk assessments of carcinogenic substances in human biomonitoring, and thus goes decisively beyond the purely descriptive statistical reference value concept. Using the presented method, quantitative dose descriptors of internal exposure can be derived from those of external exposure, provided that sufficient toxicokinetic information is available. Dose descriptors of internal exposure then allow the simple estimate of additional lifetime cancer risks for measured biomarker concentrations or, conversely, of equivalent concentrations for selected risks, such as those considered as tolerable for the general population. HBM data of chronic exposures to genotoxic carcinogens can thus be used to assess the additional lifetime cancer risk referring to the general population and to justify and prioritize risk management measures.

## 1. Introduction

In environmental health protection, human biological samples—usually body fluids such as blood, urine, or breast milk—are examined to estimate the internal exposure and the health risks of people to certain substances. To enable a standardized assessment of internal exposures, the “Human Biomonitoring Commission” (HBM Commission) of the German Environment Agency provides reference values and health-based human biomonitoring values (HBM values) for substance-specific blood, urine or breast milk concentrations [1].

The HBM Commission is an interdisciplinary and independent advisory board of scientists from academia, German research institutions, and authorities that was established by the German Environment Agency about 30 years ago to provide advice in regard to questions concerning human biomonitoring, particularly of environmental pollutants (members 2020–2023 see Appendix A).

Reference values are derived from the measured substance concentrations in human biological samples from a random sample of a defined population group using a suitable statistical method [2]. They are a measure of the background exposure in a specific time interval and are therefore not health related. In addition to these statistically defined and purely descriptive reference values [3,4,5], health-related HBM-I and HBM-II values are derived on the basis of experimental toxicological data and epidemiological studies. The HBM-I value indicates the substance concentration in human biological samples at and below which, according to current knowledge and data, no health impairments, with the exception of allergic reactions, are expected. The HBM-I value is therefore to be regarded as a test or control value.

The HBM-II value describes the concentration at and above which, in the opinion of the HBM Commission, a health impairment that can be regarded as relevant is possible. It is to be understood as an intervention and action value [1,5]. In principle, HBM values can only be derived for substances for which an exposure/dose range exists that can be assumed being harmless to health [6]. The existence of an exposure, which is harmless to health, cannot generally be assumed for carcinogenic substances.

The HBM Commission here presents its concept to evaluate the concentration of carcinogenic substances in human biological samples, which result from long-term exposure. National and international activities to establish health risk-related assessment values for genotoxic carcinogens on the basis of substance-related additional lifetime cancer risks are taken into account.

## 2. Carcinogenic Chemical Substances: Definition

Chemical substances are defined as carcinogenic if they are responsible for “(a) the induction of tumors that are not seen in non-exposed individuals; (b) the increased incidence of tumors that are also seen in non-exposed individuals; (c) the earlier development of tumors that are only seen later in unexposed individuals, or (d) the increased multiplicity of tumors” [7,8].

In its understanding of a carcinogenic chemical substance, the HBM Commission particularly follows the classification of CLP (Classification, Labelling, and Packaging) Regulation (EC) No. 1272/2008 [8], which is directly applicable and binding in their entirety for EU member states [9]. In the CLP Regulation [8], the carcinogenicity hazard class is specified in Section 3.6.1.1. as follows: “Carcinogenicity means the induction of cancer or an increase in the incidence of cancer occurring after exposure to a substance or mixture. Substances and mixtures which have induced benign and malignant tumours in well performed experimental studies on animals are considered also to be presumed or suspected human carcinogens unless there is strong evidence that the mechanism of tumour formation is not relevant for humans”.

According to the CLP, the classification of a substance as carcinogen can be further differentiated as follows: Category 1A stands for substances that are known to be carcinogenic in humans (the classification is mainly based on evidence in humans), and Category 1B for substances that are likely to be carcinogenic in humans (the classification is mainly based on evidence in animals). Chemical substances that are assigned to categories 1A or 1B are included in the list of harmonized classification and labelling of dangerous substances (Annex VI, Table 3.1 of the CLP Regulation). According to the German Hazardous Substances Ordinance [10], substances are carcinogenic if they are listed in Annex VI of Regulation (EC) No. 1272/2008 in the currently applicable version.

The HBM commission moreover regards those substances as carcinogenic that were assessed as carcinogenic in the TRGS 905 [11] as well as by the German Senate Commission for the Investigation of Health Hazards of Chemical Compounds in the Work Area [12].

Measures in the regulatory context are mainly derived from exposures to chemical substances belonging to categories 1A and 1B of the German Hazardous Substances Ordinance [10] (Carc. 1A and 1B according to the CLP Regulation [8]) or the categories 1, 2, 4, and 5 of the MAK Commission. The classification into category Carc. 2 of the CLP Regulation [8] or category 3 of the DFG MAK Commission [12] (i.e., as “suspected to be carcinogenic”) does not generally justify the initiation of measures. If toxicological data for the latter substances is available and the most sensitive endpoints are non-carcinogenic, HBM values are derived and suspicion of carcinogenicity is indicated. The need for an additional uncertainty factor or, if necessary, waiving of the HBM-I value is scrutinized and decided on a case-by-case basis.

## 3. Mode of Action

For carcinogenic substances, an exposure level that is harmless to human health, cannot generally be assumed. Whether such an effect threshold exists depends on the prevailing mode of action [13,14,15].

Chemical substances with carcinogenic properties can induce cancer development via non-genotoxic and/or genotoxic mechanisms [16,17]. The HBM Commission uses the term genotoxicity according to the definitions of the CLP Regulation (Annex 1, Part 3, Section 3.5.1.2): “The more general terms ‘genotoxic’ and ‘genotoxicity’ apply to agents or processes which alter the structure, information content, or segregation of DNA, including those which cause DNA damage by interfering with normal replication processes, or which in a non-physiological manner (temporarily) alter its replication. Genotoxicity test results are usually taken as indicators for mutagenic effects.” The direct (primary) genotoxicity of a chemical carcinogen is characterized by interaction with the DNA (intercalation, DNA adduct formation) or direct damage to the DNA (mutations by the parent substance or its metabolites), while indirect (secondary) genotoxicity is caused by processes such as oxidative stress, interference with the mitotic process, inhibition of topoisomerase, or inhibition of DNA repair [18]. A more detailed discussion of the terminology can be found in [19] and for mixtures in [20].

Non-genotoxic carcinogens, e.g., cell- and immune-damaging substances or growth stimulators (tumor promoters), develop their carcinogenic effects via a large number of other mechanisms [21]. These include cytotoxicity due to i.a. irritation, inflammation and necrosis, induced cell proliferation, receptor-mediated processes, protein binding, and hormonal effects [22]. 

Basic information on determining the mechanism of action can be found in the WHO mode of action framework for cancer and non-cancer risk assessment [23]. For carcinogenic substances in the environment that occur also in the workplace, available classifications by the DFG-MAK Commission [12] with the differentiation into categories 4 and 5 (as specified below) may provide information on the prevalent mechanism of action.

If carcinogenesis is based on direct genotoxicity, it is assumed that every molecule of a DNA-reactive substance or metabolite might trigger mutation and increase the risk of carcinogenesis. Thus, no exposure being harmless to health (no “effect threshold”) can be assumed. In this case, or if due to insufficient data no exposure harmless to health can be determined and quantified, a (usually linear) extrapolation of the experimental dose–response relationship into the low-dose range is used for risk assessment [24]. 

For non-genotoxic carcinogens it is generally assumed (for exceptions see [22]) that there is a safe exposure level below which no adverse effects occur, so that, based on corresponding evidence of the underlying mechanism of action, it is possible to deviate from a linear extrapolation into the low-dose range [25] (Section R.7.7.8.2); [26] (Section 2.2.3). For such substances, the HBM Commission has already derived HBM values, while genotoxic carcinogens are included for the first time in the HBM Commission’s approach to risk assessment of HBM data and are therefore addressed as the main focus in the sections below.

## 4. Assessment Concepts of Carcinogenic Substances in the Regulatory Context: External Exposure

In the regulatory context, various different parameters are used for a quantitative description of cancer risk.

Quantitative information for the evaluation of genotoxic carcinogens is provided, for example, by the unit risk concept of the U.S. EPA [27]. The unit risk and also the unit dose describe the estimated additional lifetime cancer risk for humans assuming lifelong exposure (usually 70 years) [28] to a unit concentration of the carcinogen, e.g., 1 µg/m^3^ air or 1 µg/L if ingested via drinking water (or after conversion to the corresponding intake levels in µg/kg bw). A *virtually safe dose* (VSD) [29] can be derived from the unit risk or unit dose and is the contaminant concentration/dose at which typically one additional cancer case occurs per 1,000,000 or 100,000 persons, i.e., risk level considered to be of low or negligible concern.

Based on appropriate animal or epidemiological studies, acceptable or tolerable concentrations for carcinogens linked to defined additional lifetime cancer risks, are proposed or legally established as a result of a risk-based assessment in the different regulatory areas. They require quantitative cancer risk estimates in the respective exposure pathway (e.g., oral or inhalation intake of the substance). For example, in regard to drinking water, the World Health Organization (WHO) determines guideline values of genotoxic carcinogens for an additional lifetime cancer risk of 10^−5^ based on quantitative exposure–risk relationships [30], whereas the EU made a policy choice for a more precautionary approach by choosing a risk value of 10^−6^ as acceptable [31]. In the Integrated Risk Information System (IRIS), the United States Environmental Protection Agency (U.S. EPA) designates discrete drinking water or air concentrations for genotoxic carcinogens for additional lifetime cancer risks of 10^−4^, 10^−5^, and 10^−6^ based on animal experimental studies and quantitative risk estimates (oral slope factor, drinking water unit risk, unit risk of inhalation exposure) [32]. The U.S. EPA’s Drinking Water Standards and Health Advisories report appropriate drinking water guidance values for an additional lifetime carcinogenic risk of 10^−4^ based on oral risk descriptors [33]. Analogous quantitative estimates of cancer risk from inhalation exposure are based on the inhalation unit risk. This approach is used by the WHO [34,35] for genotoxic carcinogens in outdoor and indoor air and gives the air concentrations for additional lifetime risks of 10^−4^, 10^−5^, and 10^−6^.

Although there is no legal definition of a tolerable or acceptable risk for carcinogens in EU legislation on chemical substances, cancer risk levels designated as ‘acceptable’ by the European Chemicals Agency (ECHA), for example, are set in the various assessment areas (cf. e. g., Annex R. 8–14 [36]). Derived minimal effect levels (DMELs) can be based on (additional) cancer risks of 10^−5^ for the workplace and 10^−6^ for the general population according to the ECHA [36]. The EFSA’s approach for the harmonized assessment of substances in foods that have genotoxic and carcinogenic properties, preferably takes (depending on the endpoint considered) the BMDL10 (benchmark dose lower confidence limit 10%) or the tumor dose 25% (T25) from an animal carcinogenicity study or—if available—a BMDL to be determined for the individual case from an epidemiological study as a reference point and determines a margin of exposure (MOE) as the ratio between the reference point and the intake by humans [26]. A MOE of ≥10,000 based on a BMDL10 from an animal study is considered by the European Food Safety Authority (EFSA) [26,37] to be of low public health concern and of low priority for justifying risk management measures.

The concept of the DFG MAK Commission for the assessment of carcinogenic substances at the workplace takes into account mechanistic aspects of carcinogenesis as well as mutagenic and carcinogenic potency [12]. The MAK Commission derives maximum workplace concentrations (MAK values) for carcinogenic agents in MAK categories 4 and 5. Category 4 focuses on a non-genotoxic mechanism of action. Category 5 classifies genotoxic carcinogens with low potency. The German legally binding risk-related measures concept of the Committee for Hazardous Substances (AGS) TRGS 910 for occupational exposure to carcinogenic hazardous substances provides an assessment of carcinogens without a tolerable effect threshold and acceptable concentrations corresponding to a cancer risk of 4:1000 and 4:100,000 (transitional 4:10,000), respectively [22].

## 5. Assessment Concepts of Carcinogenic Substances in the Regulatory Context: Internal Exposure

Concerning the assessment of an internal exposure to environmental carcinogens or carcinogens at the workplace, assessment options are also available. Biological reference values (BAR) of the DFG-MAK Commission [12] apply to a reference population of working-age individuals who are not occupationally exposed to the agent and characterize the existing background exposure to an agent [38]. For the workplace, the DFG-MAK Commission has established internal exposure equivalents for carcinogenic substances (EKA), biological workplace tolerance values (BAT values) (for the carcinogens of categories 4–5), and biological guidance values (BLW, for carcinogens of categories 1–3) [12]. In the German Hazardous Substances Ordinance, the BAT value is replaced by the biological limit value (BGW) of the Technical Rules for Hazardous Substances (TRGS 903) [39]. Substance-specific *equivalent values* in biological material for acceptable and tolerable concentrations of substances in the air at the workplace, as well as the BGW, are established by the Committee for Hazardous Substances (TRGS 910) [22].

A generalized approach to assessing carcinogens in human biomonitoring studies using *biomonitoring equivalents* (BEs) was presented by Hays et al. [40]. This early framework addresses the estimation of a BE based on animal bioassay data in combination with either animal pharmacokinetic data or human pharmacokinetic data. Typical cancer risk targets that are of interest exist within the range of 10^−6^ to 10^−4^. BEs for carcinogens to assess long-term exposure of the general population based on existing risk-related equivalent concentrations of external exposure are defined as the risk-based concentration or risk-based concentration range of a chemical or its metabolite(s), or their specific adducts in a biological matrix such as blood, urine, or another medium. BEs are derived, for example, for acrylamide [41], hexachlorobenzene [42], and benzene [43]. A similar approach was taken by Faure et al. [44] in their population-based study evaluating data for inorganic arsenic, acrylamide, and benzene from the Canadian Health Measures Survey.

## 6. The Concept of the HBM Commission for the Assessment of Carcinogenic Substances in Population-Based Human Biomonitoring

According to HBM Commission’s derivation concept, it is not possible to justify classical HBM-I and -II values, especially for carcinogens with a primarily genotoxic mechanism of action, as for these substances a safe dose cannot be defined [45]. However, a health-based assessment of HBM data of these substances is possible with a risk-based approach, similar to the assessment of external exposure. 

In contrast, in regard to carcinogens for which a threshold can be assumed, HBM-I and -II values comparable to those of non-carcinogens can be derived (e.g., for pentachlorophenol and cadmium, see [46,47]). If due to the lack of data neither a risk-based approach nor the derivation of HBM values is possible, reference values can be used at least to put the level of exposure in an orienting context.

**Figure 1:**
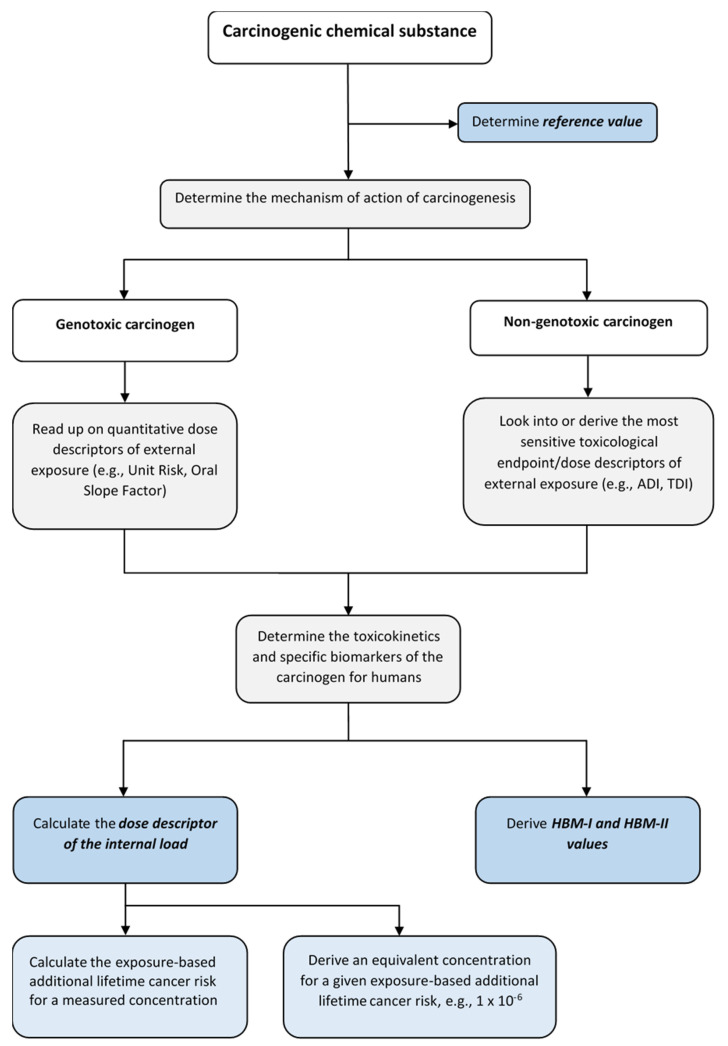
Flowchart for deriving and applying assessment values for carcinogens in population-based human biomonitoring.

The single steps of the procedure to derive and apply assessment values for carcinogens in general population-based human biomonitoring is shown in Figure 1.

### 6.1. Reference Values

Since reference values are not health based [4,5], they can also be derived for carcinogenic substances according to the criteria agreed on, irrespective of their predominant mechanisms of action, and can be used as an orienting assessment standard.

### 6.2. HBM Values for Carcinogens for Which Intake Levels Harmless to Health Can Be Derived 

Carcinogens for which a safe dose exists are treated in the same way as substances with a non-carcinogenic effect with regard to the derivation of HBM values and the corresponding recommendations for action. For these carcinogens, for which it is assumed that a non-genotoxic mechanism is the predominant mode of action, exposure levels that are harmless to health can be determined in the same way as for substances with non-carcinogenic effects [45], and thus HBM values can be derived by convention. The evaluation of these substances is based on the most sensitive toxicological endpoint of the substance, which does not necessarily have to be carcinogenicity. 

The extent to which HBM-I and -II values can also be derived for carcinogenic substances with a genotoxic mode of action that is either indirect or of very low potency is always a case-by-case decision and requires valid toxicological data. If the mechanisms of action are not sufficiently elucidated, or if the data available do not allow the safe intake level to be derived, a risk-based approach is chosen.

### 6.3. Risk-Based Approach for the Assessment of Internal Exposure to Carcinogens for Which No Safe Intake Levels Can Be Derived

For substances that induce the process of carcinogenesis via direct DNA interactions, it is generally assumed that any exposure, even the slightest, can lead to a genetic change that is carcinogenic, and thus no dose without effect can be determined. Duet to the fact that no classical effect threshold for non-carcinogenic substances can be assumed in this case, any exposure to a carcinogen with sufficient carcinogenic potency and direct genotoxicity is in consequence associated with a health risk. In this case, a health-based assessment value for internal exposure can be linked to a defined cancer risk.

According to the new concept of the HBM Commission, for the risk assessment of genotoxic carcinogens, a quantitative dose descriptor of external exposure, such as the substance-specific oral slope factor (OSF) or the inhalation unit risk (UR), must be chosen first. As the general population is the target group, vulnerable subpopulations (e.g., children, persons with underlying health conditions, and others) must be considered adequately [48].

In the absence of an adequate quantitative dose descriptor of external exposure for risk assessment of genotoxic carcinogens from widely recognized (international) organizations (e.g., EFSA, ECHA, WHO, U.S. EPA/FDA, Health Canada, and others), the dose descriptor will be derived by the HBM Commission, preferably on the basis of benchmark modeling if suitable data is available.

Then, substance-specific toxicokinetic data and suitable exposure biomarkers have to be determined.

In the next step, the toxicokinetic extrapolation of the external exposure, which is required to determine the dose descriptor of the internal exposure, is performed using the already established methodology of HBM value derivation for substances with an effect threshold, e.g., using simple compartmental models or more complex physiology-based pharmacokinetics (PBPK) modeling [6,45]. 

Finally, using the dose descriptor of the internal load, the corresponding additional cancer risk can be calculated for a measured biomarker concentration. In addition, the equivalent concentration of the biomarker for a chosen additional lifetime risk, e.g., 10^−6^_,_ is given as a comparative measure for the potency of different carcinogens. However, the classification of this additional cancer risk as potentially tolerable or acceptable has to be decided by public/political consensus and is therefore not subject of the present concept paper. 

An example for the application of this approach is given in Box 1.

Box 1 **Example for the assessment of the internal exposure to the human carcinogen benzene.**

1. **Mechanism of action:** According to the CLP regulation benzene is classified as carcinogen (Carc. 1A) and mutagen (Muta. 1B), and thus fullfills the criteria of a genotoxic carcinogen.

2. **Quantitative dose descriptor of external exposure**: The unit risk of 2.2 × 10^−6^ to 7.8 × 10^−6^ per µg/m³ is given, e.g., by the U.S. EPA [49]. To keep it clear for this example, only the unit risk of 7.8 × 10^−6^ is taken for the further calculation.

3. **Toxicokintetics**: As urinary excretion fraction (F_UE_) of inhaled benzene excreted as S-phenylmercapturic acid (S-PMA), the mean of 0.0011 is taken from Boogaard and van Sittert [50]. Furthermore, default values ^1^ of 20 m³/day inhaled air, a urine volume of 0.02 L/kg bw/day, and a bodyweight of 60 kg for the general adult population are used for subsequent calculations.

4. **Spefic biomarkers**: The urinary mercapturic acid of benzene S-PMA is taken as a very specific suitable biomarker sensitive enough for the determination of its background excretion in the non-smoking general population [51].Molecular mass S-PMA: 239.3 g/mol and benzene: 78.1 g/mol.

5. **Dose descriptor of the internal load**: In the example, the dose descriptor is determined by converting the ‘air unit risk’ for benzene into a, so to speak, ‘urine unit risk’.

An air concentration of 1 µg/m³ benzene corresponds to a *daily body dose for an adult* of 1 µg/m³ × 20 m³/d/60 kg = 0.33 µg/kg bw/d.

By means of the urinary excretion fration for S-PMA, considering the differing molecular mass from benzene and the daily urine volume per kg bodyweight, the calculated daily body dose can be converted to the corresponding *concentration of S-PMA in the urine* of 0.33 µg/kg bw/d/0.02 L/kg bw/d × 239.3 g/mol/78.1 g/mol × 0.0011 = 0.0556 µg/L.

This concentration of S-PMA in the urine corresponds to the risk of 7.8 × 10^−6^. Thus, the *dose descriptor of the internal load* can be calculated as 7.8 × 10^−6^/0.0556 µg/L = 1.4 × 10^−4^ per µg S-PMA/L ^2^.

6. **Exposure-based additional lifetime cancer risk**: For an internal exposure determined by human-biomonitoring of S-PMA in urine, the additional lifetime cancer risk can be easily calculated by using the dose descriptor of internal load, e.g., if a concentration of 0.1 µg/L S-PMA was measured in the urine, an additional lifetime cancer risk of 1.4 × 10^−4^/µg/L × 0.1 µg/L = 1.4 × 10^−5^ can be calculated.

7. **Internal load for an exposure-based additional lifetime cancer risk**: For an additional risk of, for example 1 × 10^-6^, an internal load expressed by S-PMA in urine of 1 × 10^−6^/1.4 × 10^−4^/µg/L = 0.007 µg/L can be calculated.

^1^ Default values have to be adjusted to improved approximations and considered target groups.

^2^ The dose descriptor is derived only for demonstration of the described approach and is NOT a value approved by the HBM commission.

The risk assessment for biomarkers of internal exposure to carcinogens in the general population according to Figure 1 follows in its main features the BE approach described by Hays et al. [40], but considers as a starting point for prioritization in particular the binding national and European regulatory framework of the hazard classification of chemical carcinogens. Similar to other guidelines or practical examples of deriving BEs for genotoxic carcinogens (cf. Section 5), the consideration of uncertainties for all sub-steps of the risk assessment is mandatorily established. This applies especially for the core elements *specificity of the biomarker*, *mechanism of action of the carcinogen,* as well as the *external exposure assessment value* (quantitative dose descriptor as e.g., UR, OSF). The concept of the HBM Commission is focussing on the derivation of the dose descriptor for the internal load, which does not imply a special or equivalent risk level like the BE values. Tolerable or acceptable risk levels are supposed to be set separately in the context of the risk management procedure. Thus, dose descriptors for the internal load serve to calculate both risk equivalent concentrations as well as corresponding risks for human biomonitoring data of the whole population, specifically target groups or individuals.

In the European context, the much more frequent use of HBM of carcinogens is generally observed in occupational exposure settings [52], but not for exposure of the general population. The approach of the HBM Commission provides specific guidance in particular for the risk assessment of genotoxic carcinogens in human biomonitoring of the general population. Similar to the BE values described by Hays et al. [40] substance-specific *equivalent values* in the biological material of the AGS [22] refer already to predefined acceptable and tolerable risk management concentrations of substances in the air at the workplace.

A major advantage of applying the concept presented here is that the internal exposure of the general population, in particular to genotoxic environmental carcinogens, can be assessed directly with regard to its possible relevance to health, which so far was not done by the HBM Commission. The assessment can be used, if necessary, to provide comprehensible justification for any risk management measures. The guiding risk management principle of minimizing exposure to genotoxic carcinogens as much as possible (ALARA principle) continues to apply independently of this. The precautionary principle anchored in EU legislation on chemical substances and legislation on chemical substances in the environment remains unaffected and applies in particular when the determined internal load of a specific exposure situation is above the reference values for genotoxic carcinogens.

## 7. Prerequisites for the Application of the Concept for the Evaluation of HBM Data of Genotoxic Carcinogens and Uncertainty Analysis

### 7.1. Prerequisites

The following requirements must be met in order to descriptively estimate the additional lifetime cancer risk based on HBM data of genotoxic carcinogens:

- A high specificity of the biomarker, i.e., the measured metabolite/substance or DNA and protein adducts may only be present in the human sample due to human uptake of the respective genotoxic environmental carcinogen;
- a sufficient sensitivity of the chemical analytical method;
- the availability of valid toxicokinetic data, preferably from human studies, but also from animal studies, if necessary, in order to be able to infer the (internal) biomarker concentration from repeated and long-term exposure to genotoxic environmental carcinogens;
- the availability of values of nationally/internationally established quantitative risk estimates (e.g., unit risk/unit dose) for the substance-specific cancer risk or of adequate toxicological data to derive an external dose descriptor.

The first three criteria are prerequisites that apply equally to the derivation of HBM values for *non*-carcinogenic substances from toxicological studies of external exposure or available health-based guidance values such as TDI and ADI.

For carcinogens that induce tumors due to local effects at the boundary layers of the body (e.g., skin, lung, and gastrointestinal tumors), it must be decided on a case-by-case basis whether derivation of an internal dose descriptor is appropriate.

### 7.2. Uncertainty Analysis

The HBM Commission is aware of the uncertainties in estimating and evaluating internal exposures to genotoxic carcinogens in the described approach. The identification of uncertainties and variabilities [24] within each single step of the whole process of estimating internal exposure and their impact on the risk-related assessment as a whole, as well as the description of options for their mitigation, are thus mandatory. The procedure for analysing uncertainties is presented in detail, for example, by BfR [53], EFSA [54], and WHO/IPCS [55,56].

The quality of the external dose descriptor as a measure for carcinogenic potency, often determined from animal or epidemiological studies, is of major importance. The validity of these studies as well as the adequacy of the model assumptions of the chosen extrapolation method, with respect to the resulting risk characterization, have to be assessed in detail. Population-based dose descriptors should ideally represent the population as a whole, including vulnerable sub-groups. This is, for example, not inherently the case for children, when dose descriptors from workplace studies are used and must be considered adequately. Likewise, toxicokinetic data derived from only a small number of subjects or based on animal studies must be critically examined in regard to their validity [6,45]. 

Therefore, in addition to a sound and transparent documentation of the scientific basis and the applied derivation methods of the internal dose descriptor, the detailed description of existing uncertainties is indispensable.

## 8. Conclusions

Until now, the internal exposure of the general population, or its subpopulations, to genotoxic carcinogens was quantitatively assessed within the existing range of methods of the German HBM Commission only by means of reference values. The concept presented here will now be applied as an additional assessment tool of the Commission. Due to the multi-stage assessment, a comprehensive documentation of the identified uncertainties is considered essential. Uncertainties should be communicated in an evaluated manner in the risk assessment. The concept enables profound health risk assessments of HBM findings for carcinogenic substances and thus goes decisively beyond the purely descriptive statistical reference value concept. Using the presented methodology, quantitative dose descriptors of internal exposure can be derived from quantitative dose descriptors of external exposure if sufficient toxicokinetic information is available. The latter allows for the simple estimate of equivalent concentrations for selected risks, such as those considered still acceptable for the general population. Vice versa, additional lifetime cancer risks for measured biomarker concentrations of internal exposure can be assessed to justify and prioritize risk management measures. 

## Data Availability

Not applicable.

## Appendix A. Members of the Human Biomonitoring Commission of the German Environment Agency 2020–2023

− Yvonni Chovolou, North Rhine-Westphalia Office of Nature, Environment and Consumer Protection, Recklinghausen, Germany.
− Thomas Göen, Institute and Outpatient Clinic of Occupational, Social and Environmental Medicine, Friedrich-Alexander-Universität Erlangen-Nürnberg, Henkestraße 9-11, 91054 Erlangen, Germany
− Michael Hoopmann, Governmental Institute of Public Health of Lower Saxony (Niedersächsisches Landesgesundheitsamt), Roesebeckstraße 4-6, D-30449 Hannover, Germany.
− Wilhelm Huisinga, Institute of Mathematics, University of Potsdam, Potsdam, Germany.
− Holger M. Koch, Institute for Prevention and Occupational Medicine of the German Social Accident Insurance, Institute of the Ruhr-University Bochum (IPA), Bürkle-de-la-Camp-Platz 1, 44789 Bochum, Germany.
− Andreas Kortenkamp, Brunel University London, Centre for Pollution Research and Policy, College of Health, Medicine and Life Sciences, Kingston Lane, Uxbridge UB8 3PH, UK
− Irina Lehmann, Molecular Epidemiology Unit, Berlin Institute of Health at Charité-Universitätsmedizin Berlin, corporate member of Freie Universität Berlin and Humboldt-Universität zu Berlin, associated partner of the German Center for Lung Research (DZL), Berlin, Germany
− Inge Mangelsdorf, Toxicology Consulting, Hamburg, Germany
− Claudia Röhl, Department of Environmental Health Protection, State Agency for social Services (LAsD) Schleswig-Holstein, Neumünster, Germany.
− Thomas Schettgen, Institute for Occupational, Social and Environmental Medicine, Medical Faculty, RWTH Aachen University, Pauwelsstrasse 30, D-52074 Aachen, Germany.
− Michael Schümann, Formerly Hamburg Ministry of Health and Consumer Protection, D-20539 Hamburg, Germany
− Wolfgang Völkel, Bavarian Health and Food Safety Authority, Munich, Germany
− Klaus-Michael Wollin, Formerly Public health agency of Lower Saxony, Hannover, Germany.
